# A Human Umbilical Cord Mesenchymal Stem Cell-Conditioned Medium/Chitosan/Collagen/*β*-Glycerophosphate Thermosensitive Hydrogel Promotes Burn Injury Healing in Mice

**DOI:** 10.1155/2019/5768285

**Published:** 2019-12-02

**Authors:** Panpan Zhou, Xue Li, Bing Zhang, Qing Shi, Dong Li, Xiuli Ju

**Affiliations:** ^1^Department of Pediatrics, Qilu Hospital of Shandong University, Jinan 250012, China; ^2^Cyromedicine Lab, Qilu Hospital of Shandong University, Jinan 250012, China; ^3^Stem Cell and Regenerative Medicine Research Center of Shandong University, Jinan 250012, China

## Abstract

We investigated the effects of a human umbilical cord mesenchymal stem cell-conditioned medium (MSC-CM)/chitosan/collagen/*β*-glycerophosphate (*β*-GP) thermosensitive hydrogel (MSC-CM/hydrogel) on mice with third-degree burns. MSC-CM was collected and mixed with chitosan, collagen, and *β*-GP to generate the thermosensitive MSC-CM/hydrogel, which was stored in the liquid phase at 4°C. The wounds of established third-degree burned mice were then externally covered with the MSC-CM/hydrogel, which formed a gel when placed on the wounds at physiological temperature. Injured mice in three additional groups were treated with unconditioned MSC medium (UM), MSC-CM, or UM/chitosan/collagen/*β*-GP thermosensitive hydrogels. Skin wound samples were obtained 4, 14, and 28 days after burning for further analysis by hematoxylin and eosin and Ki-67 staining. Wound healing rates and times, in addition to immunohistochemical results, were then compared and analyzed among the four groups. Application of the MSC-CM/hydrogel shortened healing time, limited the area of inflammation, enhanced reepithelialization, promoted the formation of high-quality, well-vascularized granulation tissue, and attenuated the formation of fibrotic and hypertrophic scar tissue. In summary, MSC-CM/hydrogel effectively promotes wound healing in third-degree burned mice.

## 1. Introduction

Severe burns are a common and highly lethal form of trauma and are associated with serious complications and poor prognosis [1]. The main priority for severe burn therapy is to facilitate wound healing as early as possible, and accordingly, it has been reported that mesenchymal stem cell (MSC) therapy promotes wound healing [1–4].

Most recent studies on MSC-based therapies have involved invasive operational procedures including intradermal injection of MSCs into or around the wound region and systemic intravenous injection, which contribute to patient suffering [3]. However, because of the harsh environment of the wound, the contributions of MSC differentiation to diverse ischemic injury models have been limited, including poor postimplant cell survival, engraftment efficiency, and cell retention [5]. One current hypothesis suggests that paracrine signaling from MSCs is the primary mechanism through which these cells reduce inflammation and promote angiogenesis in the wound [6, 7].

The key step in rehabilitating severe burn patients is to cover the wounds as soon as possible [8]. However, considering the intrinsic complexity of wound healing, the ideal wound dressing materials should feature good tissue conformity, a moist and occlusive environment, a low risk for infection, easy application and efficient removal of exudates, and the facilitation of healing [9]. Based on its lack of toxicity, mucoadhesion, biocompatibility, antimicrobial activity, biodegradability, and unique physicochemical properties, chitosan has received considerable attention for pharmaceutical and medical applications including drug delivery, cell encapsulation, and wound dressing [10]. GP is an organic and nontoxic compound widely used in biomedicine and has been approved by the US Food and Drug Administration [11]. Due to its physiological pH and in situ gel-forming ability at physiological temperature (considered thermosensitive), the chitosan/GP hydrogel provides an appropriate environment for bioactive agents and living cells [12, 13], while preventing migration away from the targeted administration site. Collagen, mainly collagen type I, is a major extracellular matrix (ECM) protein in the dermal tissue and plays an important role in mediating cell adhesion, migration, and proliferation owing to its specific recognition and interaction with cells. In recent years, chitosan scaffolds combined with collagen have received considerable attention and were found to have favorable chemical, physical, and biological characteristics for skin engineering, partly due to its low immunogenicity, proper porous structure, and robust mechanical properties [14, 15].

In the present study, we tested whether MSC supernatant (conditioned medium) is effective in promoting burn wound repair, as is the case for direct MSC delivery. Therefore, a thermosensitive chitosan/collagen/*β*-glycerophosphate (*β*-GP) hydrogel was used in the present study. Human umbilical cord MSCs (hUC-MSCs) are associated with outstanding advantages including short generation time, high proliferation rate, abundance, high security, convenience, and a general absence of ethical concerns compared with other sources of MSCs [16, 17].

We established a hUC-MSC-conditioned medium/chitosan/collagen/*β*-GP thermosensitive hydrogel (MSC-CM/hydrogel) and explored its efficacy for the treatment of burned skin. We aimed to maximize the concentration of MSC-associated molecules in the wound by using the hydrogel to deliver paracrine factors that are present within the conditioned media; the ultimate goal of this was to promote effective burn wound repair while providing the experimental basis for future clinical applications. To control the influence of diverse medium components, we included injured mice treated with unconditioned medium (UM) as the control group. Furthermore, mice treated with MSC-CM alone or UM/hydrogel were implemented to explore the individual effects of these components and to determine whether their combination (as MSC-CM/hydrogel) increased wound healing efficacy.

## 2. Materials and Methods

### 2.1. Preparation of Thermosensitive MSC-CM/Hydrogel

hUC-MSCs were prepared and identified as described in our previous article [18]. MSCs were obtained from umbilical cords of full-term healthy fetuses that were born via caesarean delivery in Qilu hospital of Shandong University. The use of UCs was approved by the Ethic Committee in Qilu Hospital of Shandong University, and the written informed consent from the donor was provided by the Department of Obstetrics in Qilu Hospital of Shandong University. Briefly, the umbilical cords were dissected after thorough washing, and the blood vessels were removed. The small fragments (1 mm^3^ to 2 mm^3^) were cut and placed in dishes with fresh MSC complete medium containing 90% *α*-MEM (Solarbio, Beijing, China), 10% fetal bovine serum (heat-inactivated, Gibco, BRL Co. Ltd., USA), 2 mM glutamine (Gibco, BRL Co. Ltd., USA), 100 U/ml penicillin (Solarbio, Beijing, China), and 100 mg/ml streptomycin (Solarbio, Beijing, China). After 7 to 12 days, the small tissue pieces were removed from the culture. Once the adherent fibroblast-like cells reached 80∼90% confluence, they were then trypsinized and routinely passaged at a ratio of 1 : 2∼1 : 3 for several passages. The adherent cells mainly exhibited uniform fusiform shapes, grew evenly, were arranged regularly, and were in their logarithmic multiplication cycle.

Briefly, the P3–P8 achieved cells were stained with antibodies against human antigens CD29, CD31, CD34, CD45, CD44, CD73, CD90, CD105, and CD271 (eBioscience, San Diego, CA, USA) compared with corresponding isotopes, as suggested by the manufacturer. Then, cells were analyzed by a flow cytometry system (Guava easyCyte8HT, EMD Millipore, Billerica, MA), and the data were examined with Guava Incyte (EMD Millipore). Flow cytometry results demonstrated that the UC-derived cells shared most of their immunophenotypes with MSCs, including positive stromal markers expression (CD29, CD44, CD73, CD90, and CD105) and negative hematopoietic marker expression (CD34 and CD45), endothelial cell marker CD31, and differentiated activated effector cell marker CD271. These results indicated that these cells were undifferentiated and had stem cell characteristics (data not shown). The cells we obtained were MSCs and could be used in the following experiments.

To obtain MSC-CM, upon reaching an MSC confluency of 70–80%, the medium was replaced with fresh MSC complete medium. After 24 h, the MSC-CM was collected and centrifuged at 2000 rpm for 20 min using a TDZ5-WS centrifuge (Hunan Xiangyi Laboratory Instrument Development Co., Ltd., Changsha, China) at room temperature and filtered through a 0.22 *μ*m membrane (Merck Millipore, Darmstadt, German).

A 2.2% (w/v) chitosan solution was prepared by stirring powdered chitosan (mol wt 50,000–190,000 Da; 75–85% deacetylation; Sigma, St. Louis, MO, USA) in 0.1 M aqueous hydrochloric acid (Solarbio, Beijing, China) for 3–5 h at room temperature, which was followed by sterilization by autoclaving for 20 min at 121°C [19]. *β*-GP (Merck, Darmstadt, German) was dissolved in deionized water to obtain a 50 wt% solution and then sterilized by passing through a 0.22 *μ*m filter membrane. The chitosan and *β*-GP solutions were mixed at a ratio of 5 : 1 on ice.

A sterilized 1 mg/mL collagen solution was obtained by dissolving bovine tendon collagen type I (Shanghai Jiushi Bio-Technology Co., Ltd, China) in 0.02 M acetic acid (Solarbio, Beijing, China), adjusting the pH to 7.0–7.3 with 0.1 M NaOH (Solarbio, Beijing, China), and then filtering through a 0.22 *μ*m membrane.

Finally, the thermosensitive MSC-CM/hydrogel was prepared by mixing the aforementioned precooled MSC-CM, chitosan/*β*-GP, and collagen solutions at a ratio of 1 : 2 : 1 on ice. The obtained MSC-CM/hydrogel solution was stored at 4°C for subsequent use.

After determining the pH, the obtained liquid mixture was subpackaged into test tubes and transferred to a 37°C incubator. The gelation time was determined by evaluating fluidity and viscosity using an NDJ-9S viscometer (Shanghai Performance Tai Electronic Technology Co., Ltd, Shanghai, China) [20]. In brief, the viscosity testing of MSC-CM/hydrogel mixture was performed with Rotor1^#^ and velocity of 60 rpm using NDJ-9S viscometer according to the manufacturer's protocol at different temperatures and time points, such as 4°C, 37°C 10 min, 37°C 20 min, and 37°C 30 min.

### 2.2. Determination of the Biocompatibility and Cytotoxicity of Prepared MSC-CM/Hydrogel Composites In Vitro

CCK-8 cytotoxicity tests and observations of cell morphology were performed to detect the biocompatibility and cytotoxicity of the MSC-CM/hydrogel. The procedures were performed as follows. First, MSCs in the logarithmic growth phase were applied to obtain a cell suspension of 4 × 10^6^ cells/ml in MSC-CM, which was then mixed with chitosan/*β*-GP and collagen solutions to obtain a liquid MSC-CM/hydrogel containing MSCs at a density of 1 × 10^6^ cells/ml. Subsequently, this cell-loaded hydrogel was added to a 96-well plate (40 *μ*l/well) and a 24-well plate (250 *μ*l/well) and transferred to a 37°C incubator with 5% CO_2_ for 20 min to allow gelation. Next, 100 *μ*l and 500 *μ*l of prewarmed MSC complete medium were added gently to the hydrogel in the 96- and 24-well plates, respectively. The upper medium was changed every second day. Finally, CCK-8 (Solarbio, Beijing, China) was applied in accordance with the manufacturer's protocol to evaluate the viability of MSCs in the hydrogel in 96-well plates at days 1, 3, 5, and 7; a group comprising hydrogel that was not loaded with cells was used as a blank. In addition, an equal number of routinely cultured MSCs (without MSC-CM/hydrogel) with an equal volume of MSC complete medium was prepared using the same conditions and was used as the control; MSC complete medium without MSCs was prepared as the corresponding blank. Cell morphology in 24-well plates was observed using a light microscope (IX71, Olympus, Tokyo, Japan).

### 2.3. In Vivo Wound Healing Assay Using Burned C57BL/6 Mice

All studies adhered to the procedures of the International Guiding Principles for Biomedical Research Involving Animals issued by the Council for the International Organizations of Medical Sciences and were approved by the Institutional Animal Care and Use Committee at the Shandong University Affiliated Qilu Hospital. All specimens were evaluated by two independent pathologists from the Pathology Department, Qilu Hospital, who were blinded to the treatment groups.

#### 2.3.1. Establishment and Identification of a Burn Model Using C57BL/6 Mice

Six-week-old male C57BL/6 mice were housed separately in a specific-pathogen-free laboratory and then fed for 1 week before burn model. The animals were housed with free access to water and food with controlled conditions of temperature (approximately 22°C) and relative humidity (40–60%) and a 12/12 h light/dark cycle. After anesthetizing the mice with 4% chloral hydrate (Solarbio, Beijing, China) at 10 ml/kg, dorsal hair was removed completely using clippers and a mixture of melted rosin and paraffin wax. After 24 h, the mice were anesthetized and an iron mold heated to 95°C using a temperature-controlled soldering station (Changzhou Quick Soldering Company, Changzhou, China) was placed on the hairless back for 10 s to generate a burn with a square wound area (with a side-length of 1.5 cm) [21]. Immediately, mice were sacrificed and the burn areas (complete wound with a 0.5 cm margin) were removed, fixed in 4% paraformaldehyde, and embedded in paraffin. Approximately 5 *μ*m thick sections were prepared and subjected to hematoxylin and eosin (H&E) staining in accordance with standard procedures to identify the burn grade as third-degree.

#### 2.3.2. Animal Experiment Protocol

After establishing the burn model, 72 male C57BL/6 mice (20 ± 2 g) were randomly assigned to four groups (18 mice/group) as follows: unconditioned medium (UM) (MSC complete medium) group, MSC-CM group, UM/hydrogel group, and MSC-CM/hydrogel group. UM/hydrogel was obtained by replacing MSC-CM in MSC-CM/hydrogel with UM. The hydrogels used were formulated on the day of application and stored at 4°C. Before use, the hydrogels were placed at 37°C for 20 min to gel. Wounds were debrided by removing necrotic tissue with sterile tweezers and washing with an aqueous solution of 3% hydrogen peroxide (SCR, Sinopharm Chemical Reagent Co., Ltd, Shanghai, China). Then, with a sterile cotton swab, the injured skin was covered with the corresponding UM, MSC-CM, UM/hydrogel, or MSC-CM/hydrogel, which was changed twice daily. Six mice/time point were randomly euthanized in each group 4, 14, or 28 days after creating the wounds.

#### 2.3.3. Determination of Wound Healing Rate and Healing Time

The wound size in all animals was measured daily to determine wound healing rates and to calculate the time for complete wound healing. The formula was as follows: healing rate = (original wound area − unhealed wound area)/original wound area × 100. The healing time was described as the time required for complete reepithelialization of the wound.

#### 2.3.4. Specimen Collection and Detection

*(1) H&E Staining*. Wounded skin samples from mice that were sacrificed 4, 14, and 28 days after burn induction were stained with H&E and analyzed using ImageJ 1.42q software (National Institutes of Health, Bethesda, USA). On day 4, the thickness of inflammatory infiltration, inflammatory cell density, and length of epithelial tongue were determined. The length of the epithelial tongue was defined as the distance between the advancing edges of epidermal keratinocytes and the hair follicles in normal skin tissue. On day 14, the granulation tissue thickness and fibroblast and vessel densities in the granulation tissue were measured. On day 28, the epidermal and dermal thicknesses were determined. In addition, epidermal covering [22] and dermal differentiation [23] degrees were evaluated according to the criteria summarized in Table 1.

The thickness of inflammatory infiltration and granulation tissue, epidermal, and dermal thicknesses were determined using the straight-line tool of ImageJ software, and the epithelial tongue length was measured using the segmented line tool. The density of inflammatory cells, fibroblast cells, and vessels was measured and calculated as follows: for each section, five randomized regions of inflammatory or granulation tissue were selected using the oval selection tool; within each region, the target cells and capillary lumens were counted and divided by the respective areas of the region. Values were averaged for all five regions. Three sections from each mouse per group were analyzed at each time point.

*(2) Ki-67 Staining*. Immunohistochemical staining for Ki-67 was performed on skin specimens from mice sacrificed on day 28 to evaluate cell proliferation in both the epidermis and dermis, in accordance with standard procedures. In brief, paraffin-embedded skin sections were deparaffinized, rehydrated, and then immersed in 3% hydrogen peroxide to quench endogenous peroxidase. Then the sections were blocked with 10% goat serum for 30 min followed by incubation with a rabbit anti-Ki67 antibody (1 : 100; Proteintech, Chicago, USA) at 4°C for 12 h. After washing, the tissue sections were incubated with a biotin-conjugated secondary antibody for 30 min, were visualized using diaminobenzidine, and then mounted with neutral balsam after counterstaining with hematoxylin. The cell proliferation was estimated by calculating the ratio of Ki-67-positive cells to total hematoxylin-positive cells. Three sections from each mouse per group were analyzed.

### 2.4. Statistical Analysis

All data are expressed as the mean ± standard deviation and were analyzed using SPSS 17.0 (SPSS Inc., Chicago, IL, USA). The data from two groups were analyzed by performing an independent Student's *t*-test. The data from the four animal experiment groups were first analyzed using the multiple measures analysis of variance by the Student–Newman–Keuls method and then by factorial analysis. *P* < 0.05 was considered statistically significant.

## 3. Results

### 3.1. MSC-CM/Hydrogel Owns Thermosensitive Property

**Figure 1:**
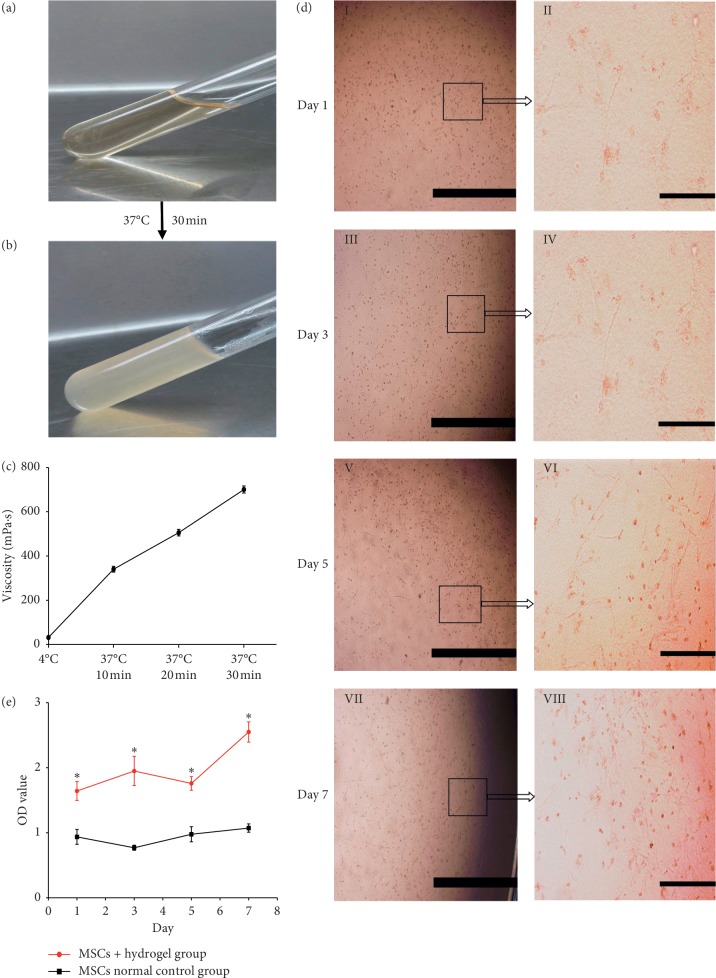
MSC-CM/hydrogel possesses thermosensitivity, with good biocompatibility and low cytotoxicity. (a) MSC-CM/hydrogel in the liquid phase at 4°C and (b) MSC-CM/hydrogel in the gel phase at 37°C. (c) A viscosity-time curve of the MSC-CM/hydrogel after transfer from 4°C to 37°C. (d) Bright-field images of MSCs cultured in MSC-CM/hydrogel on days 1 (*I*, *II*), 3 (*III*, *IV*), 5 (*V*, *VI*), and 7 (*VII*, *VIII*). Bars in *I*, *III*, *V*, and *VII* represent 2.0 mm and those in *II, IV, VI,* and *VIII* represent 500 *μ*m. (e) Assessment of biocompatibility by performing CCK-8 assays; the figure indicates absolute OD values from the CCK-8 assay performed on MSCs cultured with the MSC-CM/hydrogel and under normal culture conditions on days 1, 3, 5, and 7. The asterisk (^*∗*^) indicates *P* < 0.05 between MSCs + hydrogel and MSCs control groups at different time points.

The MSC-CM/hydrogel was a clear, transparent liquid (Figure 1(a)) with a low viscosity of 30–40 mPa·s at 4°C; however, it became an opaque, turbid, and nonfluid gel (Figure 1(b)) after incubation at 37°C for 20 min. During gelation, the viscosity increased consistently, reaching a maximum value of 650–750 mPa·s at 30 min (Figure 1(c)). The pH range of the MSC-CM/hydrogel mixture was 6.45–6.72 at 4°C before gelation and 7.0–7.19 at 37°C after gelation.

These characteristics implied that the thermosensitive MSC-CM/hydrogel could be gelatinized under physiologically relevant conditions, which suggested its potential application for in vitro and in vivo research.

### 3.2. MSC-CM/Hydrogel Exhibits Good Biocompatibility and Low Cytotoxicity

On days 1, 3, 5, and 7, the cellular morphologies of MSCs cultured in MSC-CM/hydrogel were observed. MSC-CM/hydrogel clearly promoted the spreading of embedded MSCs, which changed from a rounded morphology with little evidence of interaction with the surrounding matrix to a spindle-shaped morphology (Figure 1(d)). Results also indicated that cell proliferation was accelerated in the gel, which was confirmed by cell proliferation and toxicity experiments using CCK-8.

Cell viability was evaluated using the following formula: absolute OD value of cell viability = OD_sample_ − OD_blank_. Results showed that cell viability in the hydrogel group was significantly higher than that in the control group at each time point (Figure 1(e)). Therefore, the MSC-CM/hydrogel, with good biocompatibility and low cytotoxicity, clearly supported MSC survival and proliferation.

### 3.3. Successful Establishment of the C57BL/6 Mouse Model of Third-Degree Burns

**Figure 2:**
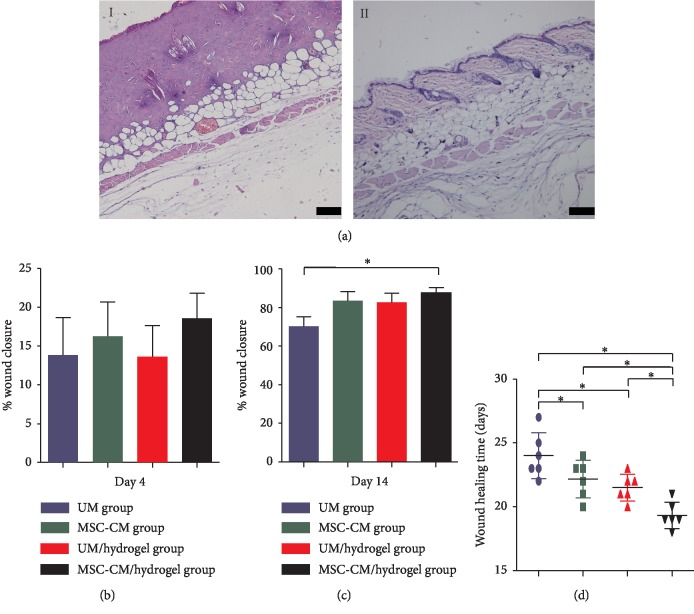
The application of MSC-CM/hydrogel promotes the wound healing of third-degree burns in mice. (a) Representative H&E staining image of established third-degree burned skin (*I*) and normal skin (*II*). Bars in A represent 100 *μ*m. Statistical analyses of wound closure rates at days 4 (b) and 14 (c), as well as wound healing time (d). The asterisk (^*∗*^) indicates *P* < 0.05 between the compared groups.

Successful induction of the burn model was achieved in C57BL/6 mice. H&E staining of burned skin immediately after the procedure showed complete destruction of the normal organization of the epidermis, dermis, and subdermal fat and muscle tissues. This result confirmed third-degree burns in this model (Figure 2(a)).

### 3.4. Considerable Efficacy of MSC-CM/Hydrogel in Wound Healing In Vivo

Among all groups, the burned surfaces of identical areas (2.25 cm^2^) at day 0 were not different, demonstrating good consistency for this model. At day 4, the healing rate in the MSC-CM/hydrogel group was not significantly higher than that in the three control groups (Figure 2(b)). However, by day 14, the healing rate in the MSC-CM/hydrogel group was significantly higher than that in the UM group (Figure 2(c)). The average healing time in the MSC-CM/hydrogel group was approximately 5 days fewer than that in the UM group and was significantly shorter than that in the UM/hydrogel and MSC-CM groups. Moreover, healing time in the latter two groups was significantly shorter than that in the UM group (Figure 2(d)).

**Figure 3:**
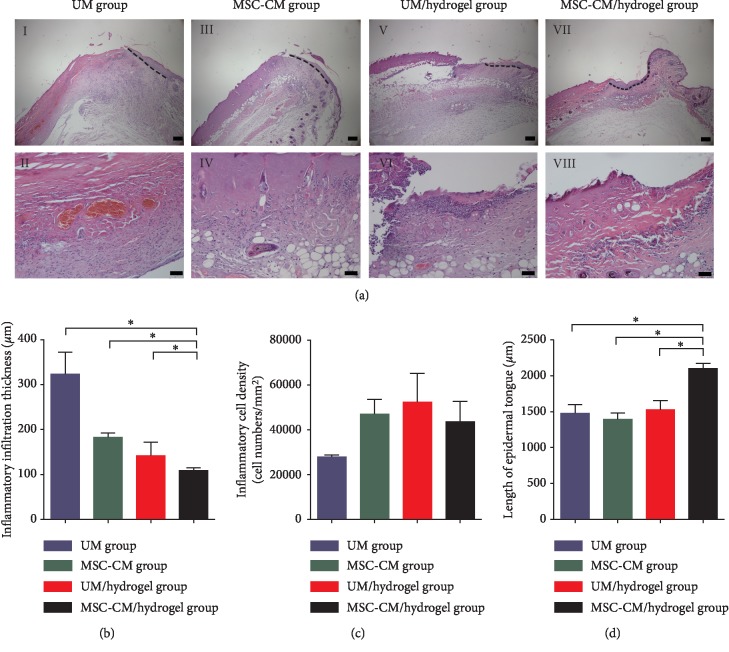
The application of MSC-CM/hydrogel suppresses wound inflammation and facilitates reepithelialization in a mouse model of third-degree burns. (a) H&E stained images of wounded skin from the four groups on day 4: UM (unconditioned medium) group *(I, II),* MSC-CM group (*III*, *IV*), UM/hydrogel group (*V*, *VI*), and MSC-CM/hydrogel group (*VII*, *VIII*). Black dotted lines in *I*, *III*, *V*, and *VII* indicate epithelial tongue lengths. Bars in *I*, *III*, *V*, and *VII* represent 200 *μ*m and those in *II*, *IV*, *VI*, and *VIII* represent 50 *μ*m. Statistical analyses of inflammatory infiltration thickness (b), inflammatory cell density (c), and length of the epithelial tongue (d) on day 4 of wound healing. The asterisk (^*∗*^) indicates *P* < 0.05 between the compared groups.

On day 4, H&E staining results confirmed the third-degree burn in all animals of four groups (Figure 3(a)). To assess the effect of the MSC-CM/hydrogel on inflammation and reepithelialization, inflammatory infiltration thickness, inflammatory cell density, and the length of the epithelial tongue were determined and compared among the four groups (Figures 3(b)–3(d)). Inflammatory infiltration thickness and the length of the epithelial tongue were significantly thinner and greater, respectively, in the MSC-CM/hydrogel group than those in the other three groups. However, there was no statistical difference in terms of inflammatory cell density among the four groups.

**Figure 4:**
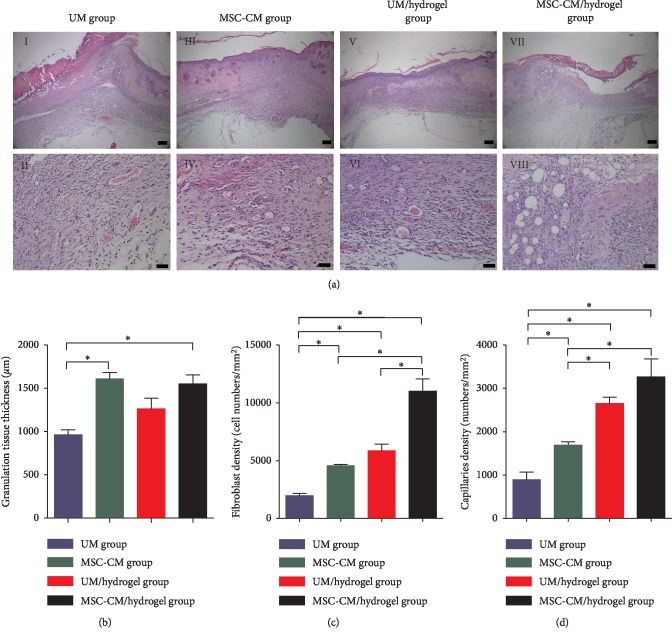
MSC-CM/hydrogel application promotes the formation of granulation tissue in a mouse model of third-degree burns. (a) H&E stained images of wounded skin in the four groups on day 14: UM (unconditioned medium) group (*I, II*), MSC-CM group (*III*, *IV*), UM/hydrogel group (*V*, *VI*), and MSC-CM/hydrogel group (*VII*, *VIII*). Bars in *I*, *III*, *V*, and *VII* represent 200 *μ*m and those in *II*, *IV*, *VI*, and *VIII* represent 50 *μ*m. Statistical analyses of the granulation tissue thickness (b), fibroblast density (c), and capillaries (d) on day 14 of wound healing. The asterisk (^*∗*^) indicates *P* < 0.05 between the compared groups.

On day 14, the granulation tissue thickness and fibroblast and capillary densities in the granulation tissue were determined and compared among the four groups to identify the effects of the MSC-CM/hydrogel on angiogenesis and the growth of granulation tissue (Figure 4). Granulation tissue in the UM group was significantly thinner than that in the MSC-CM/hydrogel and MSC-CM groups. In addition, fibroblast density in the MSC-CM/hydrogel group was significantly higher than that in the UM/hydrogel and MSC-CM groups. Furthermore, fibroblast density in the latter two groups was significantly higher than that in the UM group. Capillary density in the MSC-CM/hydrogel and UM/hydrogel groups was significantly higher than that in the MSC-CM group, whereas this parameter was significantly higher in the MSC-CM group than that in the UM group.

**Figure 5:**
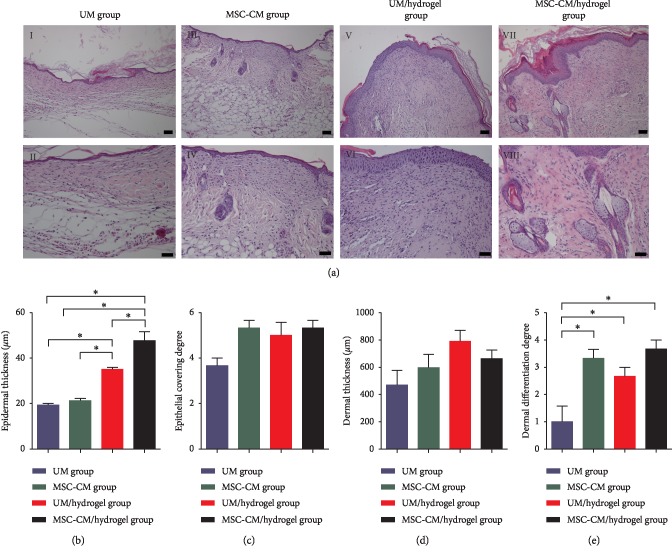
MSC-CM/hydrogel application promotes the general recovery of wounded skin in a mouse model of third-degree burns. (a) H&E stained images of wounded skin in the four groups on day 28 of wound healing: UM (unconditioned medium) group (*I*, *II*), MSC-CM group (*III*, *IV*), UM/hydrogel group (*V*, *VI*), and MSC-CM/hydrogel group (*VII*, *VIII*). Bars in *I*, *III*, *V*, and *VII* represent 100 *μ*m and those in *II*, *IV*, *VI*, and *VIII* represent 50 *μ*m. Statistical analyses of the epidermal thickness (b), degree of epidermal coverage (c), dermal thickness (d), and degree of dermal differentiation (e) on day 28 of wound healing. The asterisk (^*∗*^) indicates *P* < 0.05 between the compared groups.

On day 28, epidermal thickness, dermal thickness, degree of epidermal coverage, and dermal differentiation degree were determined and compared among the four groups to evaluate the general recovery of the wounded skin (Figure 5). Epidermal thickness in the MSC-CM/hydrogel group was significantly greater than that in the UM/hydrogel group and the latter was significantly increased compared to that in the MSC-CM and UM groups. In addition, the dermal differentiation degree in the MSC-CM/hydrogel, UM/hydrogel, and MSC-CM groups was significantly greater than that in the UM group. However, there were no statistically significant differences in terms of the degree of epidermal coverage or dermal thickness among the four groups.

**Figure 6:**
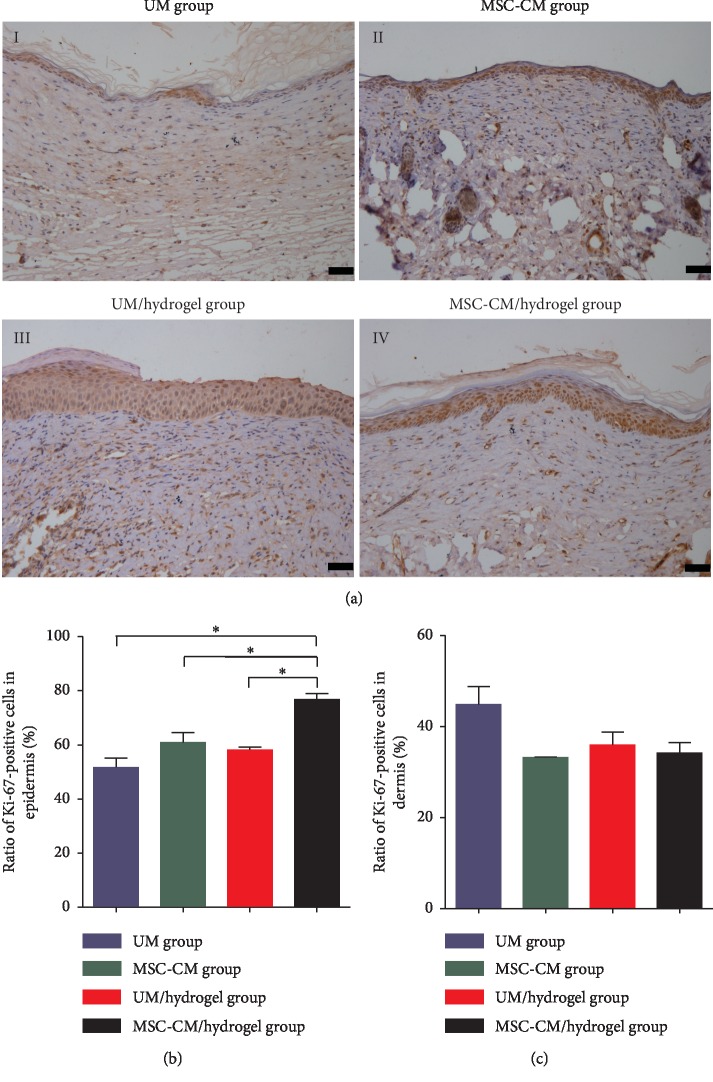
The application of MSC-CM/hydrogel promotes cell proliferation in the epidermis in repaired tissues of a mouse model of third-degree burns. (a) Ki-67 staining of repaired tissues in the four groups on day 28 of wound healing: UM (unconditioned medium) group (*I*), MSC-CM group (*II*), UM/hydrogel group (*III*), and MSC-CM/hydrogel group (*IV*). Bars represent 50 *μ*m. Statistical analyses of the proportions of Ki-67-positive cells in the epidermis (b) and dermis (c) on day 28 of wound healing. The asterisk (^*∗*^) indicates *P* < 0.05 between the compared groups.

On day 28, all mice displayed complete reepithelialization; however, wound healing remained incomplete. As the remodeling process progressed, the proliferation of cells in the epidermis (mainly keratinocytes) and dermis (mainly fibroblasts) played a vital and complex role. Ki-67 is a well-known marker of proliferation, of which expression occurs in each stage of cell proliferation (G1, S, G2, and M) but not in quiescent cells (G0). On day 28, cell proliferation in both the epidermis and dermis was detected by Ki-67 staining (Figure 6(a)), wherein Ki-67-positive cells with brown nuclei were considered proliferative. The ratios of Ki-67-positive cells in the epidermis and dermis among the four groups were calculated, analyzed, and compared (Figures 6(b) and 6(c)). The Ki-67-positive cell ratio in the epidermis of the MSC-CM/hydrogel group was significantly higher than that in the three other control groups. However, this cell ratio was not significantly different in the dermis among the four groups.

Furthermore, factorial analysis was applied to study the main effect of MSC-CM and the chitosan/collagen/*β*-GP hydrogel/mixture independently as well as their interactions. Our results (Table 2) showed that both of MSC-CM and the chitosan/collagen/*β*-GP hydrogel/mixture could promote wound healing as expected. Interestingly, the interaction of MSC-CM and the chitosan/collagen/*β*-GP hydrogel/mixture had a statistically significant main effect on day 4 epithelial tongue length and day 28 epidermal thickness (*P* < 0.05), which verifies the advantage of MSC-CM/hydrogel.

## 4. Discussion

In the present study, a thermosensitive hydrogel composed of MSC-CM, collagen, chitosan, and *β*-GP was prepared and externally applied to verify its efficacy on burn wound healing. The mechanism of gelation in the thermosensitive hydrogel involves the neutralization of chitosan by the heat-induced transfer of protons from chitosan to glycerol phosphate, which reduces the repulsive forces among positively charged ammonium groups and allows for stronger interactions among chitosan chains [24]. Physiological pH and in situ gel-forming ability at body temperature make chitosan/*β*-GP hydrogel an appropriate environment for bioactive agents and living cells and prevent its migration outside the targeted site. It is well known that hydrogels can help to deliver growth factors and cytokines, antibiotics, and cells to promote complete skin regeneration. And chitosan hydrogel could prolong retention time of delivery system on the skin surface, due to the fact that negative charge on skin surface enables electrostatically binding of chitosan to the skin surface. The hydrogel could improve the efficiency of MSC-conditioned medium by serving as a bioadhesive vehicle, prolonging the retention time of the delivery system for cytokines and growth factors [25] in wounds.

In addition to serving as a bioadhesive vehicle, chitosan itself had been proven in large numbers to promote wound healing to a certain extent; however, the mechanisms are not fully understood [26, 27]. Among those proposed are nontoxic, nonallergic, with excellent water absorption property which provides a desirable hydrated environment, promoted gas exchange, decreased inflammatory responses, increased expression of collagen and other extracellular matrix components and the formation of granulation tissue, accelerated reepitheliazation, increased formation of dermoepidermal junctions, and improved tensile strength in the healing wound. Chitosan dressing can enhance the healing process following a severe burn by direct effect on the expression of TGF*β*1. It also supports attachment and proliferation of various cell types and interacts with the growth factors released by fibroblast such as the fibroblast growth factor and enhances its bioavailability.

Besides, collagen is closely associated with cell adhesion, migration, differentiation, proliferation, and protein synthesis given on its specific recognition and interaction with cells. Therefore, collagen provides indispensable support for cell aggregation and adhesion, clot formation, and adequate scar tissue generation throughout the multistage wound healing process. Collagen-based wound dressings could accelerate wound closure and trigger a significant jumpstart to the healing process, accompanied by enhanced reepithelialization and neotissue conformation, an early angiogenic response and a significantly lower inflammatory response [28]. The presence of collagen in chitosan-collagen materials has been proved to own favorable physical, chemical, and biological characteristics for skin engineering, was associated with increased cell spreading, survival, and proliferation, and promoted gel remodeling [14, 15]. Overall, the thermosensitive hydrogel composed of MSC-CM, collagen, chitosan, and *β*-GP was expected to be more effective in wound healing than alone.

Our general observations indicated that the day 4 healing rate was highest in the MSC-CM/hydrogel group; however, this change was not statistically significant. By day 14, the healing rate was significantly improved in the MSC-CM/hydrogel group, as compared to that in the UM group, by approximately 18%. These differences could result from potential wound contraction at very early time points; however, in the last phase of wound healing, differences among all groups were clear. In addition, the shortest healing time occurred in the MSC-CM/hydrogel group, with complete healing being achieved approximately 5 days earlier, compared to that in the UM group. These results indicate that the MSC-CM/hydrogel could promote more efficient wound closure, compared to that with MSC-CM or UM/hydrogel alone.

Wound healing is a complicated biological process that occurs in three distinct yet overlapping phases including inflammation, cell proliferation, and remodeling [29]. The first stage, involving acute inflammation, occurs during the first days after burning. Burn trauma stimulates significant local and systemic inflammatory responses and induces the production of various inflammatory mediators including interferon-*γ*, tumor necrosis factor-*α* (TNF-*α*), interleukin-10 (IL-10), and IL-6 [30]. MSCs secrete key cytokines and growth factors that modulate inflammation and reduce cell death in the wound; they also produce immunosuppressive factors that suppress the proliferation of immune cells including B cells, T cells, and natural killer cells in circulation [31]. Zhang et al. [32] demonstrated that macrophages cocultured with MSCs generated low levels of the proinflammatory cytokine TNF-*α* and high levels of the anti-inflammatory cytokine IL-10, in addition to acquiring the anti-inflammatory M2 phenotype [33]. These are known as “wound healing macrophages,” which generate mediators that are essential for the resolution of inflammation and tissue remodeling, thus promoting wound repair.

The determination of inflammatory infiltration thickness indicated that, among the four groups, only the MSC-CM/hydrogel effectively limited inflammation. Although the inflammatory cell density was not statistically different among the four groups, it appeared to be higher in the MSC-CM, UM/hydrogel, and MSC-CM/hydrogel groups compared to that in the UM group. We hypothesized that the impaired migratory ability of immune cells in burns [34] could be partly redressed by the appropriate dressing. Thus, the application of the MSC-CM/hydrogel probably contributed to more localized inflammatory infiltration, resulting in a limited area of inflammation, which might have prevented an excessive inflammatory response in the surrounding tissue.

Additionally, the epithelial tongue length was significantly increased in the MSC-CM/hydrogel compared to that in the other groups. Moreover, the beneficial effects of MSC-CM/hydrogel application were increased, rather than simply being the sum of the effects of MSC-CM and chitosan/collagen hydrogel alone. It was also demonstrated that MSC-CM/hydrogel can effectively promote the migration and proliferation of epithelial cells in the burned margin area. This might be because MSC-CM contains many growth factors including epidermal growth factor (EGF) and keratinocyte growth factor (KGF) [35].

The second stage of wound repair, cell proliferation, occurs approximately 3 days to 2 weeks after injury. During this process, new blood vessels form, while sprouts of the capillaries associated with fibroblasts and inflammatory cells replace the damaged and necrotic tissue with granulation tissue. MSC-CM contains many known mediators that could regulate this process, including vascular endothelial growth factor (VEGF), basic fibroblast growth factor (bFGF), platelet-derived growth factor, EGF, transforming growth factor-*β*, and KGF [35–37]. The release of VEGF and bFGF by MSCs can facilitate the proliferation of microvascular endothelial cells, vascular stability, and the production of a long-lasting functional vascular network [38]. Moreover, the secretome of hUC-MSCs is more angiogenic than that of bone marrow derived MSCs [39]. On day 14, granulation tissue thickness in the MSC-CM/hydrogel and MSC-CM groups was significantly greater than that in the UM group. Capillary density was also significantly higher in the MSC-CM/hydrogel and UM/hydrogel groups than that in the MSC-CM and UM groups. In addition, fibroblast density in the MSC-CM/hydrogel group was significantly higher than that in the UM/hydrogel, MSC-CM, and UM groups. Thus, when considering various factors including granulation tissue thickness, new vessel formation, and fibroblast density, MSC-CM/hydrogel appeared to induce the best effects on granulation tissue formation in wounds.

The third stage of wound repair, remodeling, begins 2-3 weeks after injury and occurs for 1 year or longer. During this period, the majority of inflammatory cells, endothelial cells, and fibroblasts undergo apoptosis or exit the wound, leaving a mass containing a few cells and ECM, also known as scar formation. A scar is a nonfunctioning mass of fibrotic tissue that lacks many normal skin components including follicles, sebaceous glands, and nerve endings; this does not retain the normal tensile strength of undamaged skin [40]. The generation of hypertrophic scars is common after major injuries including burns [41]. Increased epithelial cell and fibroblast proliferation in healing tissue has been reported in hypertrophic scars [42, 43]. On day 28, in the epidermis, the MSC-CM/hydrogel group exhibited the greatest epidermal thickness and consistently had the highest Ki-67-positive ratio, which was significant compared to those in the other groups. This suggests that MSC-CM/hydrogel can promote epidermal cell proliferation better than the individual components, MSC-CM and UM/hydrogel. Surprisingly, in terms of epidermal thickness, the application of MSC-CM/hydrogel was also much more effective, rather than simply being the sum of the effects of MSC-CM and chitosan/collagen hydrogel. However, the degree of epidermal coverage in the other three groups was slightly greater than that in the UM group, but these differences were not statistically significant, which might be the result of inadequate treatment time. In the dermis, neither the thickness nor the Ki-67-positive cell ratio was significantly different among the four groups; however, it was clear that the Ki-67-positive cell ratio was lower in the other three groups than that in the UM group. Consistently, the degree of dermal differentiation in the other groups was significantly greater than that in the UM group, although significant differences were not found. The lack of significant differences might be attributed to small sample numbers, the short observation time, or the relatively fast complete reepithelialization and healing and enhanced epidermal thickness observed in the MSC-CM/hydrogel group, which impaired its effect on deeper dermal components.

Interestingly, we found that epithelial cells had a significantly enhanced proliferation rate, whereas dermal fibroblasts had a relatively lower proliferation rate in the MSC-CM/hydrogel group. Combined with the results of H&E staining, we suggest that epithelial cell proliferation is related to the multilayered, relatively well-differentiated epidermis rather than disordered growth. Moreover, the diminished fibroblast proliferation in the MSC-CM/hydrogel group indicated that this treatment did not promote hypertrophic scars. The present research also illustrated that MSC-CM/hydrogel is active and effective for tissue remodeling, with enhanced multilayering and a well-differentiated epidermis and dermis observed with this treatment. MSC therapy can transform fibrotic healing into a regenerative process, resulting in more regular epidermal and dermal structures [41]. MSCs also produce many mediators including hepatic growth factor, prostaglandin E-2 [38], IL-10, and matrix metalloproteinase-9 [44], which exert antifibrotic effects such as promoting keratinocyte proliferation [45], inhibiting myofibroblast differentiation [46], and promoting ECM turnover [47]. These effects improve wound healing functions that are specific to dermal fibroblasts [48], promoting the regeneration of ECM that more closely resembles that found in uninjured dermal tissues [49].

## 5. Conclusions

In general, MSC-CM/hydrogel application could promote cutaneous wound healing and increase the quality of regenerated skin. This treatment could shorten wound healing time, limit the inflammatory range, enhance reepithelialization, promote the formation of high-quality, well-vascularized granulation tissue, and attenuate the production of fibrotic or hypertrophic scar tissue, thereby improving wound healing rate and quality. However, the effects observed in this study were not optimal, and further efforts and modifications are required to improve the application of MSC-CM/hydrogel to burn wounds and other serious injuries.

## Figures and Tables

**Table 1:** Histological scoring of wound regeneration.

Score	Epithelial covering			Dermal differentiation
	Presence	Degree (mono- or multilayer designed)	Conical structure	
0	Absence of epithelialization	Monolayer	Not present	
1	Partial reepithelialization	Partial multilayer	Partially present	Thin, dense, and monotonous fibrosis
2	Complete reepithelialization	Multilayer	Present	Thicker but still dense and monotonous fibrosis
3				Two layers but not completely discreet
4				Two discreet layers with superficial fibrosis and loose alveolar tissue in the deep layer

**Table 2:** The result of factorial analysis.

	Main effect		The interaction
	MSC-CM	Chitosan/collagen/*β*-GP hydrogel/mixture	
Would healing time	^*∗*^	^*∗*^	
Wound healing rate: day 4			
Inflammatory infiltration thickness: day 4	^*∗*^	^*∗*^	
Inflammatory cell density: day 4			
Epithelial tongue length: day 4	^*∗*^	^*∗*^	^*∗*^
Wound healing rate: day 14	^*∗*^	^*∗*^	
Granulation tissue thickness: day 14	^*∗*^		
Fibroblast density: day 14	^*∗*^	^*∗*^	
Capillary density: day 14	^*∗*^	^*∗*^	
Epidermal thickness: day 28	^*∗*^	^*∗*^	^*∗*^
Epithelial covering degree: day 28	^*∗*^		
Dermal thickness: day 28	^*∗*^		
Dermal differentiation degree: day 28	^*∗*^	^*∗*^	
Ratio of Ki-67-positive cells in the epidermis: day 28	^*∗*^	^*∗*^	
Ratio of Ki-67-positive cells in the dermis: day 28	^*∗*^		

The asterisk (^*∗*^) indicates *P* < 0.05.

## Data Availability

The data used to support the findings of this study are available from the corresponding author upon request.
