# Correction to “A Human Umbilical Cord Mesenchymal Stem Cell‐Conditioned Medium/Chitosan/Collagen/*β*‐Glycerophosphate Thermosensitive Hydrogel Promotes Burn Injury Healing in Mice”

**DOI:** 10.1155/bmri/9835258

**Published:** 2026-04-13

P. Zhou, X. Li, B. Zhang, Q. Shi, D. Li, and X. Ju, “A Human Umbilical Cord Mesenchymal Stem Cell‐Conditioned Medium/Chitosan/Collagen/*β*‐Glycerophosphate Thermosensitive Hydrogel Promotes Burn Injury Healing in Mice,” *BioMed Research International* 2019, no. 1 (2019): 5768285, 10.1155/2019/5768285.

In the article, errors during figure preparation resulted in panels II and IV of Figure 1D being mistakenly duplicated. In addition, the indicator boxes in panels I, III, V and VII of Figure 1D were positioned incorrectly. The corrected Figure 1 is as follows:

**Figure 1:**
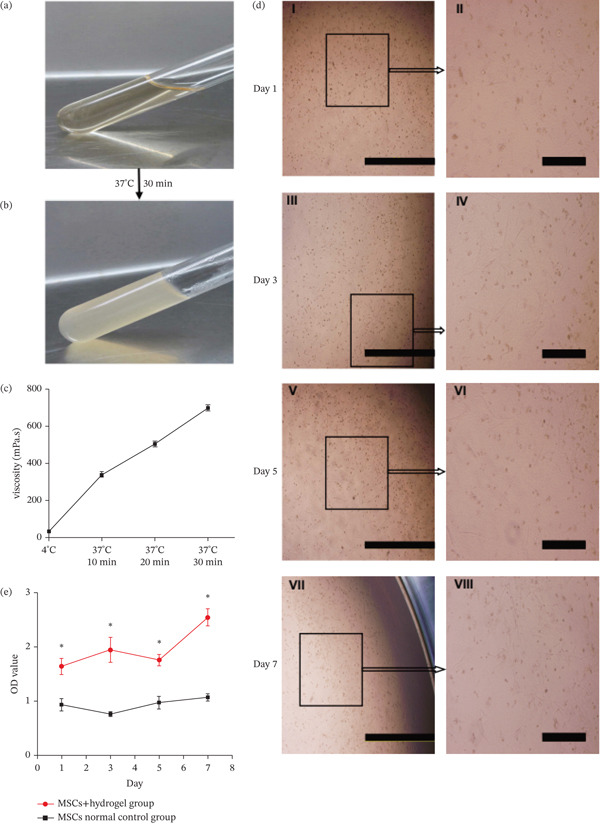
“MSC‐CM/zhydrogel possesses thermosensitivity, with good biocompatibility and low cytotoxicity. (a) MSC‐CM/hydrogel in the liquid phase at 4°C and (b) MSC‐CM/hydrogel in the gel phase at 37°C. (c) A viscosity‐time curve of the MSC‐CM/hydrogel after transfer from 4°C to 37°C. (d) Bright‐field images of MSCs cultured in MSC‐CM/hydrogel on Days 1 (*I*, *II*), 3 (*III*, *IV*), 5 (*V*, *VI*), and 7 (*VII*, *VIII*). Bars in *I*, *III*, *V*, and *VII* represent 2.0 mm and those in *II, IV, VI,* and *VIII* represent 500 *μ*m. (e) Assessment of biocompatibility by performing CCK‐8 assays; the figure indicates absolute OD values from the CCK‐8 assay performed on MSCs cultured with the MSC‐CM/hydrogel and under normal culture conditions on Days 1, 3, 5, and 7. The asterisk ( ^∗^) indicates *p* < 0.05 between MSCs + hydrogel and MSCs control groups at different time points.”

We apologize for these errors.

